# Rank-Based miRNA Signatures for Early Cancer Detection

**DOI:** 10.1155/2014/192646

**Published:** 2014-06-18

**Authors:** Mario Lauria

**Affiliations:** The Microsoft Research - University of Trento Centre for Computational and Systems Biology, Piazza Manifattura 1, 38068 Rovereto, Italy

## Abstract

We describe a new signature definition and analysis method to be used as biomarker for early cancer detection. Our new approach is based on the construction of a reference map of transcriptional signatures of both healthy and cancer affected individuals using circulating miRNA from a large number of subjects. Once such a map is available, the diagnosis for a new patient can be performed by observing the relative position on the map of his/her transcriptional signature. To demonstrate its efficacy for this specific application we report the results of the application of our method to published datasets of circulating miRNA, and we quantify its performance compared to current state-of-the-art methods. A number of additional features make this method an ideal candidate for large-scale use, for example, as a mass screening tool for early cancer detection or for at-home diagnostics. Specifically, our method is minimally invasive (because it works well with circulating miRNA), it is robust with respect to lab-to-lab protocol variability and batch effects (it requires that only the relative ranking of expression value of miRNA in a profile be accurate not their absolute values), and it is scalable to a large number of subjects. Finally we discuss the need for HPC capability in a widespread application of our or similar methods.

## 1. Introduction

A growing body of evidence is pointing to the potential role of microRNA (miRNA) profiles as biomarkers for the early detection, classification, and/or prognosis of a growing list of cancer types (for reviews of the field, see, for example, [1–3]). The miRNA profiles, obtained either from cancerous tissue or from plasma, are typically analysed for the occurrence of a signature consisting of a small set of miRNA species and having a statistically significant discriminating power. While early results are quite compelling, significant challenges remain: miRNA signatures depend greatly on the size and origin of the sample as well as on the analytical platform and protocols employed, and as a consequence the results have been found not to be always consistent.

Our signature generation and analysis method is part of a class of algorithms based on the ranking of the expression values obtained for each sample [4] and on the use of enrichment scores [5] to define a distance metric between the rank-based signatures. The combination of rank-based signatures and distance metric is used to quantify similarity between different biological states as defined by their gene expression profiles. As an example, the method described in Iorio et al. [6], MANTRA [7], was originally developed to identify and classify the pathways targeted by a chemical compound and its mode of action (MoA). According to this method, a “consensus” synthetic transcriptional response is computed for each compound summarizing the transcriptional effect of the drug across multiple treatments on different cell lines and/or at different dosages. A “drug map” is then constructed in which two drugs are connected to each other if their consensus responses are similar according to a similarity measure. The drug map is finally divided into interconnected modules using another algorithm. By analyzing these modules, the authors were able to capture similarities and differences in pharmacological effects and MoAs.

The rank-based signature method is quite general and can be applied to any phenomenon that produces a differential transcriptional signature of detectable magnitude. In a previous work we have detailed the advantages of this approach when applied to the definition and analysis of diagnostic signatures [8]. Specifically, the new method is completely agnostic about the details of the mechanisms producing the observed transcriptional response. Being rank-based and therefore insensitive in calibration errors, our method is robust to batch effects and differences in lab protocols. More importantly, it does not require a preliminary selection of a subset of genes composing the signature, a problem affecting other gene signature methods.

These benefits are particularly valuable in the analysis of circulating miRNA as biomarkers for cancer, given that the role and degree of involvement of most miRNA's in pathogenesis is still unclear and that the search for accurate biomarkers is still open for many types of pathologies. The robustness to differences in lab protocols and batch effects makes it particularly suitable for a clinical use in which such sources of confounding signals are often unavoidable.

In this paper we present a new application of our method and make a case for its suitability for large-scale use, for example, as a mass screening tool for early cancer detection or for at-home diagnostics. The novelties with respect to previous works are in (i) the application of the method (large-scale use), (ii) the type of diagnosis to be performed (early detection of cancer), and (iii) the type of data used as input (circulating miRNA). These aspects are all connected. For example, in the case of mass screenings for the early detection of cancer, the tool needs to be minimally invasive (hence the use of circulating miRNA), must have demonstrated efficacy for this specific application (we show the results achievable on a real dataset), and must be scalable to a large number of subjects (we discuss the scalability of the analysis of the diagnostic maps produced by the method).

## 2. Description of the Rank-Based Signature

The conceptual novelty that we introduce is a notion of sample-specific signature, coupled with a metric to quantify the degree of similarity between any two such signatures. Contrary to current practices, instead of evaluating expression profiles by means of a common yardstick (a single list of highly discriminating RNA species), our method first seeks to summarize the characteristics of each sample employing a specific signature, and then it performs a systematic, all-to-all signature comparison. The expected result is the emergence of a partitioning of the set of samples in separate groups on the basis of signature similarity. The classification problem is then reduced to the simpler task of identifying the phenotype associated with each of the resulting groups, in what we call the labeling step. In order to facilitate the partitioning step, a similarity map of the samples is constructed in the form of an undirected graph, in which samples are represented as nodes and sample-to-sample edges are drawn with a length inversely proportional to the degree of mutual similarity. After applying our method to a large number of expression profile sets, we have indeed observed a marked tendency of nodes to spontaneously cluster into clearly visible groups. The relatively simple final labeling step can be carried out using one of many possible empirical methods, for example, by noting the membership of a few additional samples whose classification is known a priori.

**Figure 1:**
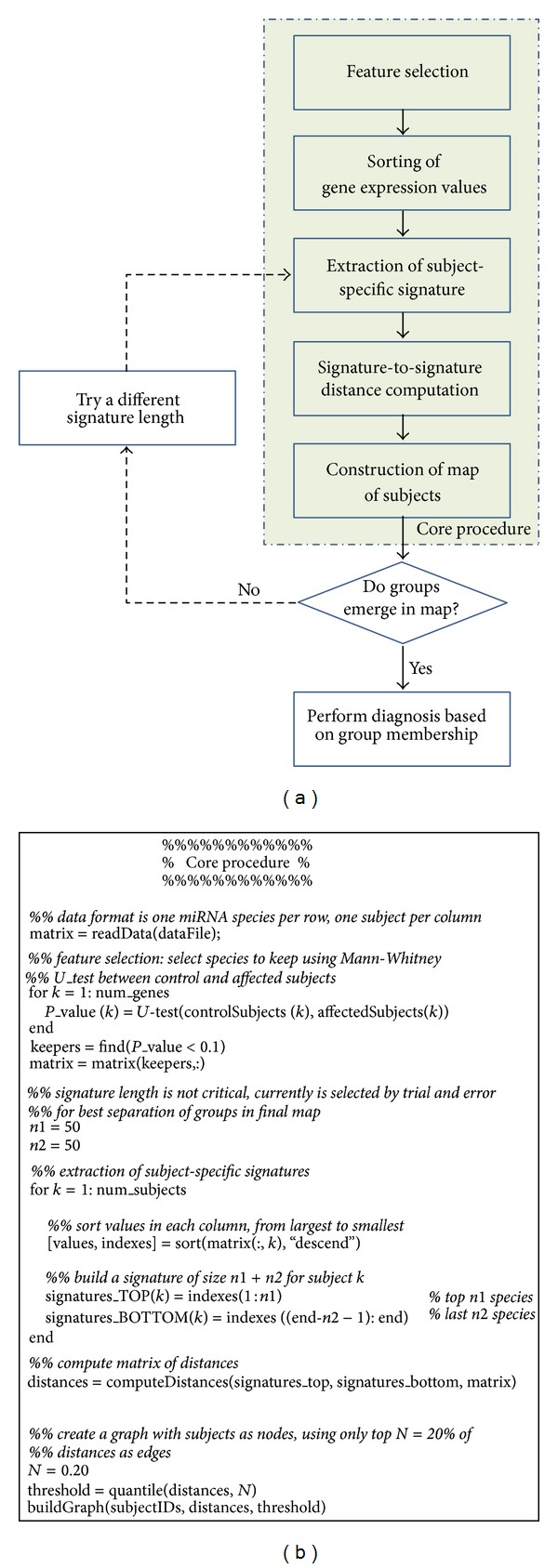
Wiring diagram of our overall diagnostic signature method (a) and pseudocode of the core procedure (b).

The base of our method is the definition of signature associated with each expression profile, where by profile we refer to the set of miRNA concentration values produced by a miRNA microarray or by a RT-PCR quantification assay. As a first step, we rank the values of the selected genes from the highest to the lowest. The signature is represented by the set of first *n*1 and the last *n*2 miRNA species IDs (or probe IDs) in the ranking; in other words, our composite signature is composed by the identity of the *n*1 most expressed and *n*2 least expressed genes. The values of *n*1 and *n*2 are two input parameters of our method (see variables *n*1 and *n*2 in the pseudocode of Figure 1(b)) and are currently selected by hand. For a detailed discussion on the meaning and role of the algorithm parameters, the reader is referred to our previous work [8]; in the same work we show that the value of these parameters is not critical; that is, the procedure is robust with respect to the choice of parameter values.

In all the experiments described in this paper we perform two preprocessing steps on the data. The first is the computation of the transformation of each input profile into a differential one with respect to a virtual control profile, and the second is a selection of miRNA species to be considered for the signature definition. The virtual control profile is computed as the average of all the input profiles. By dividing each input profile by the virtual control, we transform it into a differential profile (not shown in Figure 1), in order to increase the sensitivity of our method to deviations of expression values from the norm.

As a second step, we perform what is called a feature selection operation on the list of miRNA species, using a relatively low threshold (i.e., resulting in a rather loose selection). In other words, we apply our method not to all the probes in the array, but only to the subset of those showing statistically significant differences between control and affected groups, as determined by a Mann-Whitney *U*_test with a nonstringent significance level (*P*_value = 0.1). Interestingly, this preselection step was not required with early generation miRNA microarrays containing 400–700 probes but has become a necessity with current generation arrays having typically in excess of ~1000 probes. Since our method has been shown to work well with the 20 K+ probes of typical DNA microarrays without any preselection, we attribute the need for this extra step to the diluting effect of the large percentage of control probes added in latest generation miRNA microarrays.

The pseudocode, reported in Figure 1(b), succinctly describes the essential steps of the algorithm for defining the signatures and computing the matrix of their reciprocal distances, starting from a set of array data in the form of a matrix (one probe per row, one array per column). The output of the code is a distance matrix in the form of an edge list, which includes all the distances below a user-selected threshold value.

The distance between each pair of samples is computed by (i) finding the enrichment score (ES) [5] of the signature of one sample against the whole list of sorted expression values of the other sample (i.e., ES of sample A signature against list of values of B), then (ii) finding the ES obtained by inverting the role of the two samples (i.e., ES of sample B signature against list of values of A), and finally (iii) averaging the two scores. Since a signature is composed of two parts, top and bottom, the ES of a signature is the average of the ES of its two parts computed separately against the other sample (i.e., (ES_top_ + ES_bottom_)/2).

In the map construction step, the resulting distances are employed to draw a map of the testing set samples with the help of a tool such as Cytoscape [9]. The map is in the form of a graph, with each node representing a sample and edges of proportional length representing distances. Only the smallest *N*% of distances are selected and thus represented as edges of corresponding length in the map; typically, the *N* = 10% shortest distances are sufficient to produce a clear map.

The analysis of the resulting map represents the last and crucial step of the method. Most of the time this map can be easily partitioned in clusters or modules (group of nodes with a higher percentage of connections to internal nodes than to external ones, also referred to as communities) by hand; if however the clusters are not easily delineated, the partitioning can be performed with the help of one of the many unsupervised community identification algorithms described in the literature (i.e., GLay method in the clusterMaker plugin of Cytoscape [10]). Additionally, the core steps leading to the map production (signature extraction, distance measurement, and map drawing) can be repeated using different values of the input parameters *n*1, *n*2, *N*, in order to increase the cluster separation in the resulting map. Once a satisfactory partitioning is achieved, the relatively simple step of assigning a control/affected phenotype label to each cluster can be performed according to a number of empirical methods, for example, by adding a few labeled samples from the training dataset or inspecting the sign of the expression change of disease genes, that is, genes known from the literature to be associated with the condition under study. The overall procedure is illustrated by the diagram in Figure 1(a).

## 3. Rank-Based Signatures as Early Breast Cancer Biomarkers

A necessary condition for the use of any transcriptional signature as biomarker for cancer is that the healthy versus cancer miRNA transcriptional profiles are sufficiently divergent to produce a map of practically relevant discriminatory value. A number of recent results have examined the validity of this hypothesis for a number of cancer types. The work by Zhao et al. [11] provides some strong evidence in the case of early stage breast cancer. Using microarray-based expression profiling followed by real-time quantitative polymerase chain reaction (RT-qPCR) validation, the authors compared the levels of circulating miRNAs in plasma samples from 20 women with early stage breast cancer (10 Caucasian American (CA) and 10 African American (AA)) and 20 matched healthy controls (10 CAs and 10 AAs). Using the significance level of *P* < 0.05 constrained by at least twofold expression change as selection criteria, they found that 31 miRNAs were differentially expressed in CA study subjects (17 up and 14 down) and 18 miRNAs were differentially expressed in AA study subjects (9 up and 9 down). Interestingly, only 2 differentially expressed miRNAs overlapped between CA and AA study subjects.

While the work by Zhao et al. is a genome-wide study, it focuses on identifying single miRNAs as biomarkers. Our work instead is based on a complex signature as a biomarker and, in this respect, is closer to the work by Boeri et al. [12] on circulating miRNAs as biomarker for lung cancer. The differences with respect to Boeri et al. approach are the following: (i) our signature is derived from the profile of all miRNAs present in a chosen platform (miRNA microarray), (ii) we use a robust rank based definition of signature, and (iii) we introduce an intuitive notion of distance between signatures to draw a map of the samples and graphically represent their degree of similarity in terms of spatial closeness.

We analysed the expression data described in the Zhao et al. paper (GEO accession GSE22981); as done in the Zhao paper, we analysed the AA and the CA group separately. The profiles in the dataset were obtained with the Illumina Human v2 MicroRNA Expression BeadChips platform, were used as downloaded from the GEO repository, and included 1134 miRNA species after removing those with missing values. Of these, the feature selection step (*P*_value = 0.1) selected 184 species for the AA group and 316 species for the CA group. Using our method, we measured the reciprocal distances between the twenty samples in each group and then we drew a map based on such distances. The values of the signature size that gave the best results in terms of separation of the two groups in the final graph were *n*1 = *n*2 = 25 for AA and *n*1 = *n*2 = 50 for CA. For the sake of clarity we only represented distances falling below a suitably chosen threshold (*N* = 20%, meaning we used the smallest 20% distances), as large distances are obviously less interesting. The map is in the form of undirected graph and was drawn using Cytoscape; the layout is the one produced by Cytoscape with the force-directed method (force is interpreted as 1-distance).

**Figure 2:**
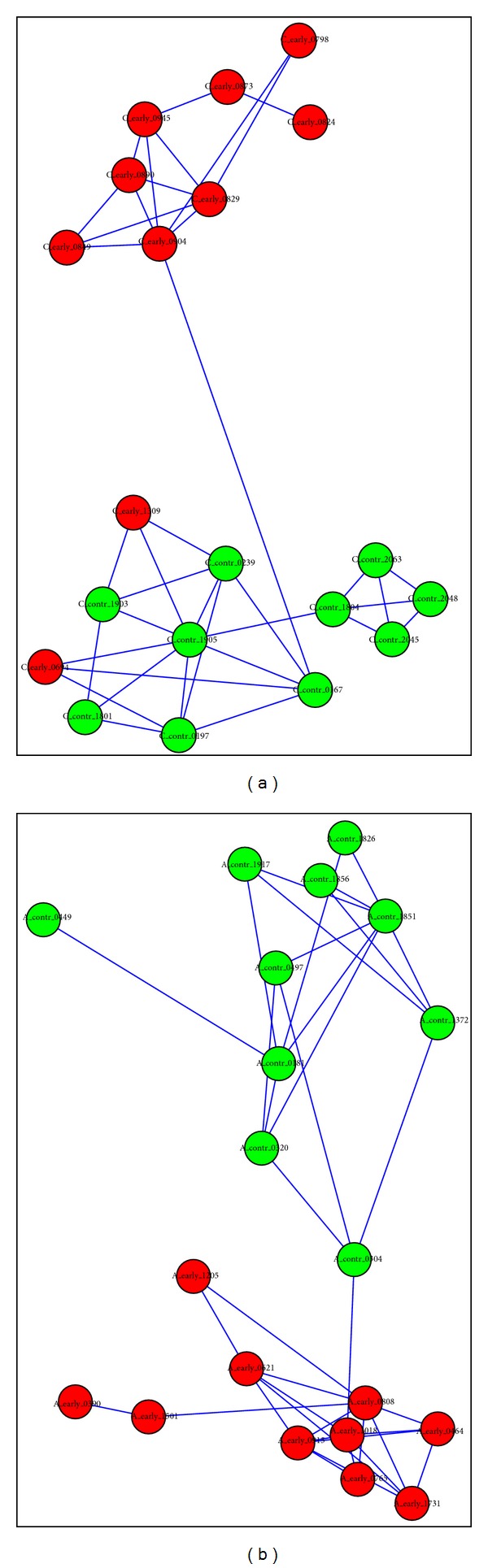
Maps for the two groups of subjects: (a) Caucasian subjects and (b) African American subjects. Each node in the graph represents a subject, whose transcriptional signature was derived from a profile of circulating miRNA. The length of an edge is approximately proportional to the inverse of the distance between two signatures as computed by the algorithm. The nodes spontaneously cluster in well-defined and easily identifiable control/disease groups, with only two misclassifications in the Caucasian subjects case. Node label legend:* red* = early breast cancer subject;* green* = control subject.

It can be seen from Figure 2 that the signatures end up in easily identifiable control/disease clusters; only two samples in the CA patient group end up in the wrong group resulting in a wrong diagnosis. Therefore, based on this limited sample we conclude that our diagnostic tool has 100% specificity and 90% average sensitivity (80% to 100% depending on the cohort. Comparison with the Zhao results is not straightforward: while they achieve at best 70% sensitivity at the 100% specificity level using microarray data, their best result is 90%/100% sensitivity/specificity but only for one patient group (AA) and using RT-PCR data.

## 4. Rank-Based Signatures as Early Lung Cancer Biomarkers

**Figure 3:**
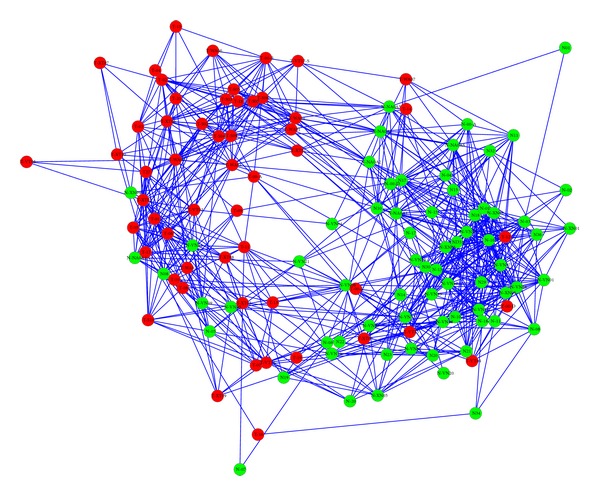
Map of patients based on their serum miRNA profiles included in the Bianchi et al. dataset [13]. We applied our method using a signature length of 25 + 25 and the top 10% quantile as threshold for distances (*N* = 10%). Using a neighbor majority rule, the accuracy of the classification is 84%. Node color legend:* green* = healthy subjects;* red* = early stage subjects.

In order to quantify the predictive power of our method as biomarker of early cancer we tested it on the dataset from the paper of Bianchi et al. [13]. In such paper the authors describe a state-of-the-art diagnostic test to identify asymptomatic high-risk individuals with early stage lung cancer based on serum circulating miRNA. The dataset is composed of serum miRNA profiles of 124 patients of which 70 were healthy and 54 were asymptomatic patients diagnosed with early stage lung cancer using low dose spiral computed tomography (LD-CT). The biomarker identified by the authors consists of a collection of 34 miRNA species selected for their discriminating power; for our method, we selected a signature length of 25 + 25 (i.e., 25 most expressed and 25 least expressed species) and a *N* = 10% threshold for distances (i.e., only top 10% is used to draw the map). The profiles included 141 miRNA species, of which 51 were selected in the feature selection step. In the Bianchi et al. paper the dataset was divided in two subsets, used for training and testing, respectively; we treated the profiles as a single set. The resulting map is shown in Figure 3, where the red and green nodes represent at-risk and healthy patients, respectively.

As described before, the diagnosis for a new subject can be obtained by adding his/her profile to the input dataset and noticing in which group the new node is going to fall. The one shown in Figure 3 is an example of a map in which the control/affected groups, while still clearly visible, are not as neatly separated as the one in the previous study (see Figure 2), and therefore reaching a diagnosis for a new subject might be not such an obvious task. In such a case a diagnosis can still be performed by selecting an algorithmic rule on how to make a healthy/affected call for each node/patient. A simple and intuitive rule is to look at the label of the immediate neighbor nodes and perform a majority count; a more sophisticated version of the rule would weigh the votes according to the inverse of the neighbor distances. By using the unweighted count (in which a tie is counted as an erroneous diagnosis), we obtain 84% accuracy, compared with the 78% and 80% accuracy on the training and testing sets, respectively, reported by Bianchi et al. This level of performance might be improved with further tuning of the algorithm; we are also working on a version that automatically selects the length of the signature, which should increase the ease of use of the algorithm and its sensitivity.

## 5. Diagnostic Signatures in the Context of a Mass Screening Programme and Implications for HPC

Our new approach to biomarker definition has been recently validated in an open international competition, the SBV IMPROVER Diagnostic Biomarker Challenge, organized by IBM Research and Philip Morris International to evaluate and compare state-of-the-art approaches to the identification of diagnostic biomarkers based on gene expression profiles [14]. In the course of the challenge our novel biomarker demonstrated its validity and generality by producing top scoring diagnostic signatures for a set of diseases as diverse as lung cancer, multiples sclerosis, psoriasis, and chronic obstructive pulmonary disease; our team ranked 2nd overall and 1st in the MS subchallenge out of 52 participants. Overall, the single most important lesson from the competition was that it is possible to reliably predict phenotype from genomics data. This is an important contribution given that recently the use of gene expression data for phenotype classification has been questioned [15] or has been discredited due to poor practices [16].

One major advantage of diagnostic signatures based on expression profiles with respect to existing practices is that a test based on circulating miRNA biomarkers is inherently much less invasive than a mammography or a colonoscopy. Furthermore the method described here for blood miRNA is also being tested on urine and saliva miRNA, making it not only even less invasive, but also potentially suitable for self-administration once a suitable simplified quantification/profiling technology has been developed. The twist is that the result of a test, in the form of a miRNA profile for a certain individual, needs to be interpreted by comparing it with a number of other (known) profiles in order to produce a diagnosis. Therefore, it is easy to envision a scenario in which dedicated on-line repositories maintain both the clinical and the transcriptomic data of millions of individuals. Such repository would provide the real-time interpretation of newly produced miRNA profiles as a service, while ensuring compliance with the current privacy regulations on the handling of sensitive data. The necessity of an HPC capability to provide such service is compound by the fact that expression profiles might be compared to several reference datasets (i.e., collections of profiles of subjects with certified diagnosis), each relative to a type of cancer or of other disease, and that such datasets will be dynamically updated based on advances of our understanding of subtypes of cancer, their treatment, histological classification, and so forth.

The signature definition and analysis algorithm presented here is not particularly computationally intensive as long as the analysis is restricted to a modest number of samples (i.e., up to a few hundreds profiles). The execution times for the datasets described in the previous sections (~40 profiles of ~1 K miRNA species) are on the order of 10 seconds, using GNU Octave 3.6.4 [17] on a Windows 7 laptop with a 1.3 GHz SU4100 CPU and 8 GB of RAM, and go up to about 15 minutes for a dataset of ~100 mRNA profiles obtained with gene expression microarrays (~20 K probes), such as the ones used in the SBV IMPROVER Diagnostic Biomarker Challenge [18].

However, the possibility of using the algorithm as a part of a mass screening program for the early detection of cancer would require scaling up its execution capability by several orders of magnitude. Such scaling up would be needed to accommodate (i) the potential number of subjects tested (10^3^ to 10^6^), times (ii) the collection of certified profiles used for the reference map (10^2^ to 10^3^), times (iii) the types of cancers to be tested for (10^0^ to 10^2^), times (iv) the number of times an individual will be tested in his/her lifetime (10^1^ to 10^2^). The HPC platform selected for the task would have to meet not only the computational requirements but also the storage requirements needed for holding and making available online the large body of reference datasets plus the individual medical data. Such levels of hardware performance are comparable to those currently achieved by large search engines like Google and therefore might adopt the same large-scale server farm architecture [19].

An important observation regarding the genetic versus genomic view of personalized medicine is that any expression profile-based biomarker essentially produces a diagnosis by taking a good look at a snapshot of the transcriptome of an individual taken at a certain point in his/her life. This is different than taking a look at the genome of an individual, as gene expression is comparatively much more dynamic compared to the genome. During a life of an individual cells accumulate genomic changes as a consequence of the various kinds of stress they are subject to; however, not all of them have phenotypic consequences. Environmental influences and lifestyle changes also bring modifications to gene expression and add to the complexity of the picture. The analysis of the transcriptome at different time points in life provides a “downstream” view of all these influences and can reveal which pathologies are actually developing at those instants. Given the highly dynamic nature of most types of cancers and their dependence on the complex interplay of a wide range of causes (genetic, environmental, and lifestyle), it would be advisable to periodically perform a diagnostic test like the one described here, generalizing the preventive approach already being implemented in current screening campaigns for the early detection of breast and colon cancers.

## References

[1] Jay C, Nemunaitis J, Chen P, Fulgham P, Tong AW (2007). miRNA profiling for diagnosis and prognosis of human cancer. *DNA and Cell Biology*.

[2] Reid G, Kirschner MB, van Zandwijk N (2011). Circulating microRNAs: association with disease and potential use as biomarkers. *Critical Reviews in Oncology/Hematology*.

[3] Schwarzenbach H, Hoon DSB, Pantel K (2011). Cell-free nucleic acids as biomarkers in cancer patients. *Nature Reviews Cancer*.

[4] Tan AC, Naiman DQ, Xu L, Winslow RL, Geman D (2005). Simple decision rules for classifying human cancers from gene expression profiles. *Bioinformatics*.

[5] Subramanian A, Tamayo P, Mootha VK (2005). Gene set enrichment analysis: a knowledge-based approach for interpreting genome-wide expression profiles. *Proceedings of the National Academy of Sciences of the United States of America*.

[6] Iorio F, Bosotti R, Scacheri E (2010). Discovery of drug mode of action and drug repositioning from transcriptional responses. *Proceedings of the National Academy of Sciences of the United States of America*.

[7] MANTRA. http://mantra.tigem.it/.

[8] Lauria M (2013). Rank-based transcriptional signatures: a novel approach to diagnostic biomarker definition and analysis. *Systems Biomedicine*.

[9] Smoot ME, Ono K, Ruscheinski J, Wang P-L, Ideker T (2011). Cytoscape 2.8: new features for data integration and network visualization. *Bioinformatics*.

[10] Morris JH, Apeltsin L, Newman AM (2011). ClusterMaker: a multi-algorithm clustering plugin for cytoscape. *BMC Bioinformatics*.

[11] Zhao H, Shen J, Medico L, Wang D, Ambrosone CB, Liu S (2010). A pilot study of circulating miRNAs as potential biomarkers of early stage breast cancer. *PLoS ONE*.

[12] Boeri M, Verri C, Conte D (2011). MicroRNA signatures in tissues and plasma predict development and prognosis of computed tomography detected lung cancer. *Proceedings of the National Academy of Sciences of the United States of America*.

[13] Bianchi F, Nicassio F, Marzi M (2011). A serum circulating miRNA diagnostic test to identify asymptomatic high-risk individuals with early stage lung cancer. *EMBO Molecular Medicine*.

[14] Tarca AL, Lauria M, Unger M (2013). Strengths and limitations of microarray-based phenotype prediction: lessons learned from the IMPROVER diagnostic signature challenge. *Bioinformatics*.

[15] Ioannidis JPA (2005). Microarrays and molecular research: noise discovery?. *The Lancet*.

[16] Goozner M (2011). Duke scandal highlights need for genomics research criteria. *Journal of the National Cancer Institute*.

[17] Eaton JW, Bateman D, Hauberg S (2009). *GNU Octave Version 3.0.1 Manual: A High-Level Interactive Language for Numerical Computations*.

[18] Rhrissorrakrai K, Rice JJ, Boue S (2013). sbv IMPROVER diagnostic signature challenge: design and results. *Systems Biomedicine*.

[19] Barroso LA, Dean J, Hölzle U (2003). Web search for a planet: the google cluster architecture. *IEEE Micro*.

